# Identification of Single-Nucleotide Polymorphisms (SNPs) Associated with Heat Tolerance at the Reproductive Stage in Synthetic Hexaploid Wheats Using GWAS

**DOI:** 10.3390/plants12081610

**Published:** 2023-04-10

**Authors:** Ambreen Mehvish, Abdul Aziz, Birra Bukhari, Humaira Qayyum, Zahid Mahmood, Muhammad Baber, Muhammad Sajjad, Xuequn Pang, Fenglan Wang

**Affiliations:** 1 Institute of Molecular Biology and Biotechnology, Bahauddin Zakariya University, Multan 60800, Pakistan; 2 College of Life Sciences, South China Agricultural University, Guangzhou 510642, China; 3 International Maize and Wheat Improvement Center (CIMMYT) Pakistan Office, National Agriculture Research Center (NARC), Park Road, Islamabad 44000, Pakistan; 4 Department of Plant Sciences, Quaid-i-Azam University, Islamabad 45320, Pakistan; 5 Institute of Crop Sciences, National Agriculture Research Center (NARC), Park Road, Islamabad 44000, Pakistan; 6 Department of Biosciences, Comsats University, Islamabad 45550, Pakistan; 7 College of Horticulture and Landscape Architecture, Zhongkai University of Agriculture and Engineering, Guangzhou 510408, China

**Keywords:** synthetic hexaploid wheat, association mapping, heat tolerance, heat shock proteins, quantitative trait nucleotides

## Abstract

The projected rise in global ambient temperature by 3–5 °C by the end of this century, along with unpredicted heat waves during critical crop growth stages, can drastically reduce grain yield and will pose a great food security challenge. It is therefore important to identify wheat genetic resources able to withstand high temperatures, discover genes underpinning resilience to higher temperatures, and deploy such genetic resources in wheat breeding to develop heat-tolerant cultivars. In this study, 180 accessions of synthetic hexaploid wheats (SHWs) were evaluated under normal and late wheat growing seasons (to expose them to higher temperatures) at three locations (Islamabad, Bahawalpur, and Tando Jam), and data were collected on 11 morphological and yield-related traits. The diversity panel was genotyped with a 50 K SNP array to conduct genome-wide association studies (GWASs) for heat tolerance in SHW. A known heat-tolerance locus, *TaHST1*, was profiled to identify different haplotypes of this locus in SHWs and their association with grain yield and related traits in SHWs. There was a 36% decrease in grain yield (GY), a 23% decrease in thousand-grain weight (TKW), and an 18% decrease in grains per spike (GpS) across three locations in the population due to the heat stress conditions. GWASs identified 143 quantitative trait nucleotides (QTNs) distributed over all 21 chromosomes in the SHWs. Out of these, 52 QTNs were associated with morphological and yield-related traits under heat stress, while 15 of them were pleiotropically associated with multiple traits. The heat shock protein (HSP) framework of the wheat genome was then aligned with the QTNs identified in this study. Seventeen QTNs were in proximity to HSPs on chr2B, chr3D, chr5A, chr5B, chr6D, and chr7D. It is likely that QTNs on the D genome and those in proximity to HSPs may carry novel alleles for heat-tolerance genes. The analysis of *TaHST1* indicated that 15 haplotypes were present in the SHWs for this locus, while hap1 showed the highest frequency of 25% (33 SHWs). These haplotypes were significantly associated with yield-related traits in the SHWs. New alleles associated with yield-related traits in SHWs could be an excellent reservoir for breeding deployment.

## 1. Introduction

Wheat is a major staple food for almost 40% of the global population, cultivated on almost 220 million hectares of the land, with 750 million tons of production and a trade value of almost USD 50 billion per year [1]. Wheat yields are highly restricted by high temperatures and are prone to becoming more variable with the changes in the environment. Wheat is highly susceptible to high-temperature stress during anthesis and is less prone to recuperate if stressed at this critical stage. In wheat, for anthesis, the optimal temperature ranges between 5 °C and 28 °C. Temperatures that exceed this optimal range reduce the grain yield and quality by reducing the seed set and grain filling [2]. It is anticipated that every 1 °C rise in temperature causes a 6% decrease in the global wheat yield [3]. Important physiological and biochemical processes of the plant are interrupted by heat stress. The knowledge of heat stress’s effects and tolerance at the physiological, morphological, and biochemical levels is very important for making new crop varieties that can cope with the upcoming climatic changes [4].

Genetic diversity in major food crops, including wheat, has been successfully manipulated via conventional breeding approaches, resulting in a 0.8–1.2% annual genetic yield gain over the last 100years. However, the abovementioned gain will not be enough to meet the increasing global demand of food by 2050 [5]. Therefore, a gradual increase in genetic gain is necessary to cope with the demands of the 2% annual rise in the global population. The narrow genetic diversity in bread wheat—especially within the D-genome—needs to be extended to introduce new alleles for productivity improvement. To address this issue, synthetic hexaploid wheat (SHW) has been artificially synthesized [6]. SHW is produced artificially by creating a fertile hybrid between diploid wild goat grass (*Aegilops tauschii*, D’D’) and tetraploid durum wheat (*Triticum turgidum*, AABB). For more than three decades, the CIMMYT (the International Maize and Wheat Improvement Center) has made and used SHW to overpass gene shifts from durum wheat and *Ae*.*tauschii* to bread wheat (hexaploid). This is an exclusive example of success in exploiting wild relatives in conventional breeding at a huge, global scale [7]. SHWs are valuable for the enhancement of wheat in areas where stress due to high temperatures occurs more often [8]. 

Genome-wide association studies (GWASs) have been shown to be an effective method for discovering genes that underpin complex phenotypes. The genetic basis of heat tolerance in wheat has also been studied using GWASs. Heat stress tolerance (HST) is a very complex process and is controlled by many genes. Bread wheat contains a large and complex genome (~17 Gb) with more than 85% repetitive sequences [9]. Due to the complex nature of heat stress, very few genes for the response to heat stress in wheat have been cloned [10,11]. Advances in molecular marker development and quantitative genetics have made it possible to discover quantitative trait loci (QTLs) that regulate HST in wheat. Using specific features as indicators, many QTLs with significant effects on HST have been discovered [11,12]. Bennet et al. [13] identified two major QTLs on chr3B by using a set of 255 double-haploid (DH) lines; these QTLs show significant effects on canopy temperature and grain yield. Using a recombinant inbred line (RIL) (Halberd × Cutter), Mason et al. identified a significant QTL on chr3B related to the heat susceptibility index and yield components [14]. Maulana et al. [15] identified different significant QTLs in bread wheat at the seedling stage. QTLs for spike length were identified on chr2A, chr3B, and chr7D. For leaf chlorophyll content, multiple QTLs were identified on chr2B, chr2D, chr4B, and chr5B. QTLs were identified for seedling recovery on chr2A, chr2B, chr2D, chr3A, chr7B, and chr7D. Several other GWAS experiments have been conducted to dissect heat tolerance in wheats from the US [15], Pakistan [16], the CIMMYT [17], China [18], and emmer-derived hexaploid wheat [19].

The *TaHST1* locus is present on chr4AL in bread wheat [20]. This region of 0.949Mb has proven to be essential for heat stress tolerance at the seedling and reproductive stages. Nineteen genes were identified in this region as playing a significant role in HST. Partial or complete deletion of this region was detected in most of the bread wheat cultivars. The presence of this region significantly enhanced the HST in wheat. Deletion of this region affected the grain number, grain weight, grain length, and thousand-grain weight [20].

This study was designed to (i) characterize a diversity panel consisting of SHWs against HS at the reproductive stage, (ii) identify quantitative trait nucleotides (QTNs) associated with HST at the reproductive stage in synthetic hexaploid wheat accessions, and (iii) characterize the *TaHST1* locus and identify haplotype variation associated with heat stress at the reproductive stage.

## 2. Results

### 2.1. Phenotypic Data Analysis

Phenotypic data showed significant variation among all traits in both conditions (control and HS). The analysis of variance (ANOVA) for all of the morphological traits is presented in Table S2. All of the traits showed significant variations for genotypes, genotype × location, genotype × year, genotype × treatment, and other possible combinations, with the exception of genotype × year interaction for some traits (e.g., PH and TKW). 

The ambient environmental variables—such as daily maximum temperature (T_max_), daily minimum temperature (T_min_), daily average temperature (T_avg_) in °C, and relative humidity (%) during the field trials—are described in Table S3. For example, among all locations, Sindh received the hottest days (>30 °C) compared to Bahawalpur and Islamabad. The descriptive data presented in Table 1 show the variations in SHWs under control and HS conditions. In control conditions, the mean DH was 119 days, with a range from 102 days (AUS33402) to 157 days (AUS34259), while in HS conditions the mean DH was 89 days, with a range from 77 days (AUS33403, AUS33415, AUS33421, AUS34453, and AUS34458) to 115 days (AUS34257). 

In control conditions, the mean value for GY was 9.4 t/ha, with a range from 7 t/ha to 12 t/ha, while in HS conditions the mean value for GY was 6 t/ha, with a range from 4 t/ha (AUS30672, AUS33386, AUS33397, AUS33404, AUS34235, and AUS34257) to 8 t/ha (AUS30627, AUS30642, AUS30648, AUS33414, and AUS34231). For TKW in control conditions, the mean value was 47 g, with a range from 19 g (AUS30660) to 68 g (AUS33417), while in HS conditions the mean value was 36 g, with a range from 18 g (AUS34257) to 49 g (AUS30296). Phenotypic data are presented in Supplementary Table S2.

**Figure 1:**
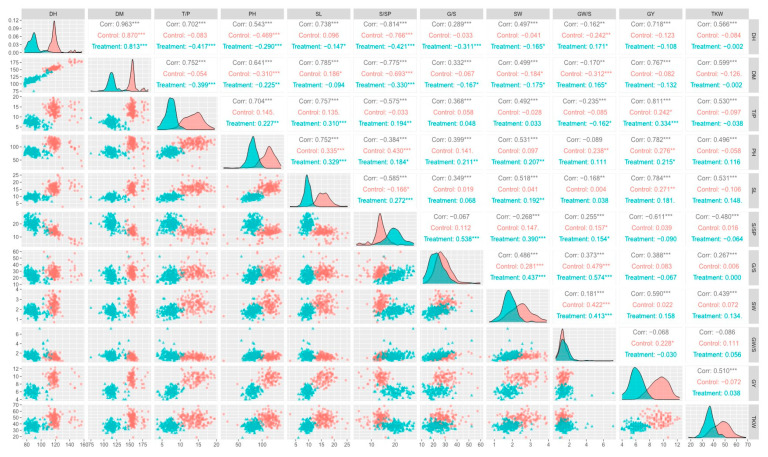
Pearson’s correlation coefficients (r) among multiple traits in control and HS conditions. *, **, *** represent significant p-values at *p* < 0.05, 0.01 and 0.001 respectively.

Pearson’s correlation coefficients are given in Figure 1 for all traits in control and HS conditions, along with their distribution graphs. Positive correlation was observed between yield-related traits such as DH and TKW (r = 0.566 ***), GY and DH (r = 0.718 ***), SW and SL (r = 0.518 ***), PH and TKW (r = 0.496 ***), SW and GY (r = 0.590 ***), GY and TKW (r = 0.510 ***), GY and SL (r = 0.784 ***), and SL (r = 0.752) and DM and SL (r = 0.785 ***). 

Similarly, most of the other traits showed significant positive correlation with one another. Some of the traits showed negative correlation with one another, i.e., SpSP showed highly negative correlation with DH (r = −0.814 ***), DM (r = −0.775 ***), TpP (r = −0.575 ***), PH(r = −0.384 ***), SL (r = −0.585 ***), GpS (r = −0.067), SW (r = −0.268 ***), GY(r = −0.611 ***), and TKW (r = −0.480 ***).

### 2.2. Marker Statistics

In total, 66,836 markers were genotyped using the 50 K SNP chip. Thirty-eight markers were removed after quality control due to missing data. A further 27,485 markers were removed due to having MAF < 0.05. Subsequently, 39,313 markers were used for the GWASs. These SNPs were distributed on all chromosomes, with the maximum number of SNPs on chr6A (*n* = 2484) and the minimum number of SNPs on chr4D (*n* = 794). The B-genome contained the greatest number of SNPs (*n* = 15,190), followed by the A-genome (*n* = 14,269) and the D-genome (*n* = 9926). 

### 2.3. QTNs Associated with Yield Traits under Heat Stress

In total, 143 QTNs were identified, out of which 48 QTNs were identified in control conditions, 52 QTNs were identified in HS, and 43 QTNs were identified for HSI. The greatest number of QTNs was identified for GpS (*n* = 24), while the fewest QTNs were identified for SpSP (*n* = 6). On chr2B and 5B, the highest number of QTNs (*n* = 12) was identified, while the lowest number of QTNs (*n* = 1) was found on chr5A and chr7A. In total, two QTNs were consistently identified in control and HS conditions. A QTN on chr7B at ~437 Mb was consistently associated with DM in control and HS conditions. Another QTN on chr6A at 390 Mb was consistently associated with DM both in control and HS conditions. 

**Figure 2:**
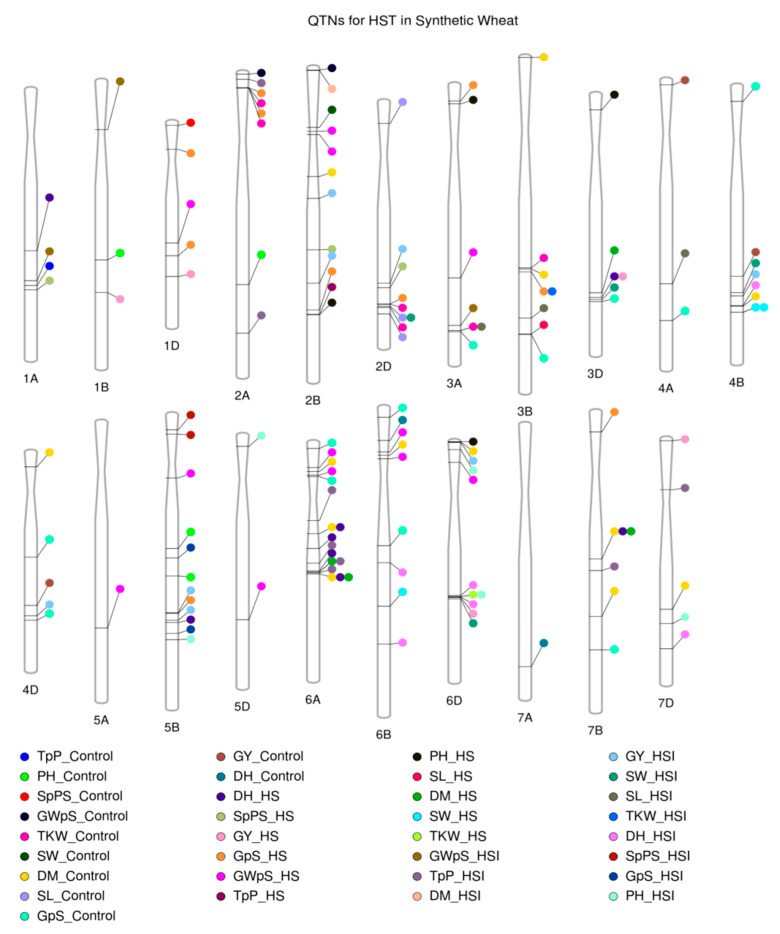
Location of QTNs identified on the chromosome. Abbreviations: DH: days to heading; DM: days to maturity; TpP: tillers per plant; PH: plant height; SL: spike length; SpSP: spikelets per spike; GpS: grains per spike; SW: spike weight; GWpS: grain weight per spike; GY: grain yield; TKW: thousand-kernel weight; HS: heat stress.

In total, 15 QTNs were pleiotropically associated with multiple traits. A QTN on chr1A at ~560 Mb was associated with multiple yield-related traits, including GWpS, TpP, and SpSP, in both control and HS conditions. A QTN on chr2A at ~ 44 Mbs was associated with GpS and TKW in both HS and control conditions. On chr2B, a QTN found at ~6 Mb was associated with GWpS and DM in both HSI and control conditions. Another QTN on the same chromosome at ~731 Mb was associated with TpP and PH under HS conditions. A QTN on chr2D at ~598 Mb was associated with GpS and TKW in both HS and control conditions; another QTN on the same chromosome at ~ 606 Mb was found to be associated with SL and SW in control and HSI conditions. A QTN was found on chr3A at ~ 727 Mb, associated with SL and TKW in both HSI and control conditions. On chr3B, three QTNs were found to be associated with multiple yield traits: one at ~625 Mb, associated with TKW and DM in control conditions; another at ~637 Mb was associated with GpS and TKW in both HS and HSI conditions; and a QTN at ~822 Mb was associated with SL and GpS in both HS and control conditions. A QTN was found on chr3D at ~602 MB, linked with DH and GY in HS conditions. On chr5B, a QTN was found at ~588 Mb, associated with GY and GpS in both HS and HSI conditions. On chr6A, two QTNs were found: one at ~270 Mb, associated with DH and DM in both HS and HSI conditions; and the other at ~380 Mb, associated with DH, TpP, and DM in both HS and HSI conditions. A QTN on chr6D at ~458 Mb was linked with DH, PH, TKW, and GY in both HS and HSI conditions. On chr7B, a QTN at ~437 Mb was found to be associated with DH and DM in both HS and control conditions. All of the QTNs associated with multiple traits in HS conditions are presented in Figure 2 and Table 2.

Likewise, the QTNs associated with multiple traits were designated as pleiotropic QTNs (Table 3). In total, 14 pleiotropic QTNs were identified, out of which 3 QTNs on chr2D, chr3D, and chr6D were contributed by the D-sub genome and may carry novel alleles from *Ae. tauschii*. A QTN on chr2A at ~44 Mb was associated with GpS and TKW under both HS and control conditions. Another QTN on chr3D was associated with DH and GY and could be an important locus because plants tend to flower early to escape heat stress, and simultaneous control of such traits is an important finding. 

### 2.4. Co-Localization of QTNs and Heat Shock Proteins

In total, eight QTNs were found in proximity to HSPs under HS conditions (Table 4). Two QTNs on chr2B were found in proximity to HSPs, but the QTN (AX-94760904) associated with TpP was the closest to *TaHSP70.24*, at ~731 Mb. A QTN (AX-95237322) on chr3D at ~45 Mb associated with PH was found in proximity to HSP TaHSP40.95. A QTN (AX-109911103) on chr5A at ~611 Mb and a QTN (AX-94886530) on chr5B at ~186 Mb, associated with GWpS, were found in proximity to HSPs *TaHSP40.150* and *TaHSP40.155*, respectively. 

**Figure 3:**
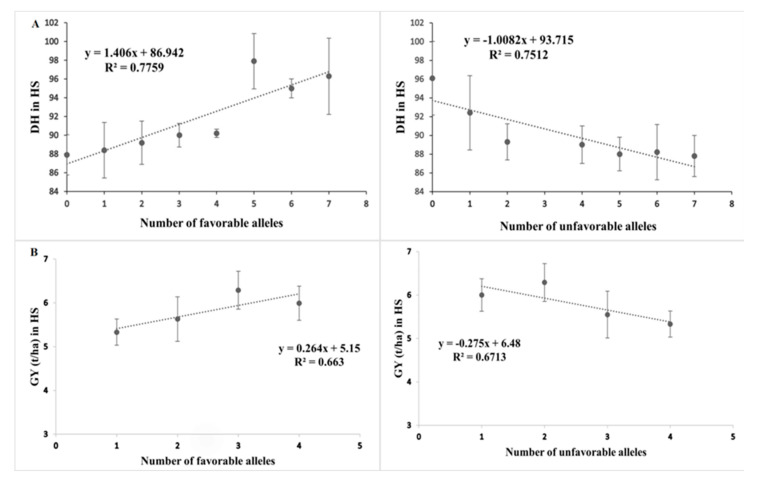
Scatterplot showing the effects of favorable and unfavorable alleles on the yield traits DH (days to heading) and GY (grain yield). In (**A**) the effect of number of favorable alleles and unfavorable alleles on DH in HS condition is shown and in (**B**) the effect of number of favorable and unfavorable alleles on the grain yield is shown.

Based on the SNP effect, favorable and unfavorable alleles were identified, and their frequencies were determined. For DH, seven favorable and unfavorable alleles were identified in HS conditions. The scatterplot in Figure 3 shows the effects of the favorable and unfavorable alleles on DH; with the increase in the number of favorable alleles, the number of days to heading decreased significantly, while an increase in the number of unfavorable alleles reduced the DH. A similar pattern was observed in GY: with the increase in the number of favorable alleles, GY increased, while with the increase in the number of unfavorable alleles, GY decreased. The coefficients of determination (R^2^) indicated that effect of favorable alleles ranged from R^2^ = 0.77 (DH) to R^2^ = 0.66 (GY) (Figure 3), while for unfavorable alleles the range of effect was from R^2^ = 0.75 (DH) to R^2^ = 0.67 (GY) (Figure 3).

### 2.5. Characterization of the TaHST1 Locus in the Diversity Panel

**Figure 4:**
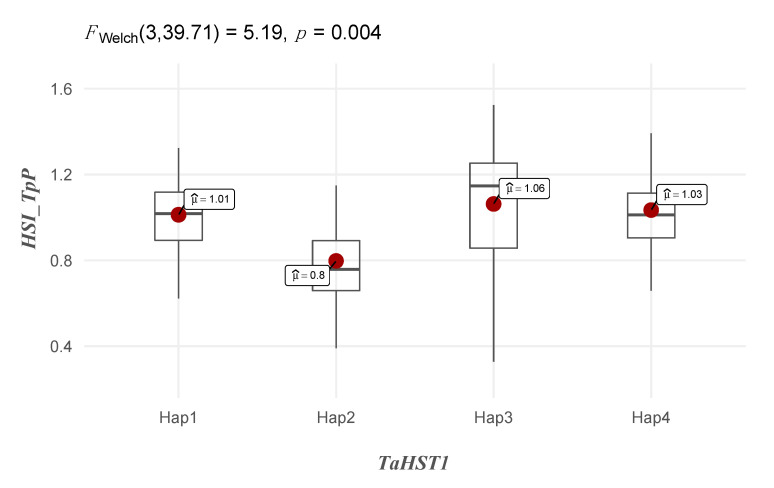
Phenotypic effects of the top four *TaHST1* haplotypes on the heat susceptibility index for tillers per plant in synthetic hexaploid wheats.

Characterization of the *TaHST1* locus in synthetic wheat accessions was performed using PCR-based primers. Based on the results of these primers, 15 haplotypes were identified. Hap-1 showed the highest frequency, at 25%, and all markers showed positive amplification. Hap-2 was present in 16% of accessions, and only two markers showed positive amplification, while Hap-3 and Hap-4 were present in 15% and 10% of accessions, with one and two deleted sites, respectively. The effect of top Hap1 to Hap4 was significant on HSI for TpP (Figure 4). All of the haplotypes, along with their frequency and phenotypic data under HS, are presented in Table 5.

## 3. Discussion

### 3.1. Phenotypic Variation for Agronomic Traits in SHWs under Heat Stress

In the past, a small number of SHWs and their derivatives were characterized to explain why they are superior to the conventional bread wheat genotypes, and they were evaluated for high-temperature tolerance [22,23,24,25]. Here, we describe the performance of a larger collection of 180 SHWs, which were sown at the normal time and late to characterize their variation in many agronomic traits and their relationships under heat stress conditions. ANOVA illustrated significant variation of accessions under normal and heat stress conditions, and the outcomes were consistent with those previously documented for bread wheat when identifying the genotype by environmental interactions and considerable variance in agronomic traits [26,27]. A greater variation was shown by SHWs for all of those traits that could be exploited in breeding for heat tolerance [25]. Overall, the highest broad-sense heritability was observed for DH, indicating that this trait has a high response to selection, and under HS conditions the large variations observed in SHWs could be very useful in heat tolerance breeding programs for wheat germplasm to fight high-temperature stress. Early heading and prematurity are preferable for high yield gains under heat-stressed conditions, due to the stress avoidance mechanism. 

As expected, the GY of SHWs was reduced significantly under HS conditions. Conversely, late heading and late maturity resulted in low GY, demonstrating that the enhanced production is a result of their adaptation and capability to avoid late heat stress. To avoid the late high-temperature stress by the end of the season, early heading and maturity enable the SHWs to fill their grains casually. PH showed high-to-moderate heritability estimates and could also be a useful trait in HS tolerance breeding. To bring improvement in wheat, GY has always been the most important criterion for direct breeding, and the further improvement of GY through direct selection may not be easy, due to the low heritability estimates suggested by the findings of the present study. The low heritability estimates obtained for GY suggest that direct selection of the GY for further betterment may be difficult in wheat. Therefore, indirect selection for increased GY at elevated temperatures using yield-related traits is an appropriate criterion for screening for tolerance to abiotic stresses, including heat and drought.

### 3.2. Effects of Heat Stress in Synthetic Hexaploid Wheat

It was observed that under HS conditions, all of the traits showed a decreasing trend, except for SpSP. This is likely due to the fact that spikes emerge at the onset of the reproductive stage when temperature conditions are optimal. Some SHW accessions (e.g., AUS30284, AUS30286, AUS30297, AUS30637, AUS30645, and AUS30660) showed higher resilience to HS conditions for yield-related traits.

GY is highly affected by the environment, as described by Sharma et al. [28]. During heat stress conditions, a noticeable decrease in yield and other developmental traits was observed, and many other reports of the heat stress responses of wheat also drew the same conclusion [28]. High-temperature stress has shown significant decreases in PH [29], maturity time [30], flowering time [31], number of grains per spike and weight of grains per spike [32], TKW [33,34], and GY [35]. The reduction in GY was observed more during the year 2015 as compared to 2014 (39% and 36%, respectively), because of higher temperatures. During this experiment, we observed that the 2 °C rise in temperature in the year 2015 reduced the GY by 5% compared to 2014. A previous study observed a 3 to 4% decrease in wheat production under controlled conditions for every 1 °C increase above 15 °C, and the number of kernels declined by 12.5% for every 1 °C increase from 25/20 °C to 35/20 °C [33]. Similarly, it has been indicated by global simulations that the production of wheat will decrease by 6% on average for every 1 °C rise in temperature, equating to a yield decline of almost 42 million tons [36].

### 3.3. GWASs for the Identification of Loci Associated with Heat Stress Tolerance

Identification of QTNs associated with HST is crucial for breeders. Therefore, a number of gene mapping studies were conducted at the flowering and reproductive stages [13,37]. Recently, Tanin et al. [21] compiled all of the abiotic-stress-related QTLs in wheat as meta-QTLs and provided a detailed framework for comparative studies. In total, 11 meta-QTLs were aligned with those found in our work, including 3QTLs for GY on chr1B, chr1D, and chr2B. Two QTLs were identified by Sangwan et al. [38] on chr2A and 2D for DH. In our study, no QTNs were identified on chr2A or2D. However, eight QTNs were identified on chr1A, chr3D, chr5B, chr6A, and chr7B in HS. Similarly, two QTLs were identified on chr2D for DM [38]. In contrast, four QTNs were identified in our study on chr3D, chr6A, and chr7B. For PH, four QTNs were identified on chr2B, chr3A, chr3D, and chr6D. Meanwhile, in previous studies, two QTLs were identified on chr2A and chr4A for PH [38]. For SL, three QTLs were identified by Maulana et al. [15] on chr3B and chr7D. 

In our studies, a QTN was identified on chr3B for SL in HS. Three QTNs were identified for SpSP on chr1A, chr2B, and chr2D. No QTL had been identified previously for SpSP on these chromosomes. In contrast, eight QTLs were identified on chr1D, chr3A, chr4B, chr4D, chr5A, chr6A, and chr6B [39]. Three QTLs were identified on chr2B, chr3A, and chr5B for GpS [40]. In our study, three QTNs were identified on same chromosomes for GpS in HS. For GY, two QTLs were identified on chr2B and chr2D [41]. In our studies, five QTNs were identified on chr1B, chr1D, chr3D, chr6D, and chr7D for GY in HS. For TKW, in our study, only one QTN was identified on chr6D in HS, while five QTLs were identified by Li et al. [40]—on chr1D, chr4D, chr5A, chr5D, and chr6B. As discussed by Ogbonnaya et al. [42], favorable alleles in a single environment show little improvement in multiple-stress environments. We also observed a similar phenomenon in the present study, where environment-specific MTAs and MTAs for the average environments did not significantly overlap. Differences in loci controlling stability and environment-specificity suggest that there may be separate evolutionary trajectories for them.

### 3.4. Co-Localization of QTNs and HeatShock Proteins

All of the HS-specific QTNs in proximity to HSPs could be ideal candidates for genes underpinning heat tolerance, and due to the use of SHWs they are likely to carry new alleles of HSPs. Several HSPs (e.g., *TaHSP100, TaHSP70*, and *TaHSP90*) are induced underheat stress conditions [26]. In this context, the QTN for SW on chr4B is likely to be a good candidate for further studies, because its closet HSP is TaHSP70.52, which was reported to be induced during heat stress. There is a need for detailed analysis of the HSPs reported in this study for further validation. 

### 3.5. Characterization of the TaHST1 Locus in the Diversity Panel

The *TaSHT1* locus plays a significant role in HST at both the seedling and reproductive stages. Five markers were used to detect the presence of the*TaSHT1* locus in SHW [17]. Recently, Khan et al. (2022) [43] identified 24 haplotypes of *TaHST1* in Pakistani wheat cultivars. Fifteen haplotypes were determined based on the results of these markers. The results indicated that the presence of this QTL has significant effects on wheat under heat stress conditions. Two markers for this locus (Xhau-2 and -3) have shown significant associations with phenotypic traits under heat stress conditions. It is likely that haplotype distribution in SHW is different from that in cultivated wheats, and that new alleles are available due to the use of durum wheat in developing SHWs. The combination of 127 bp alleles at Xhau-4 and the presence of alleles at Xhau-5 have a positive impact on yield-related traits and could be used as a positive marker for selecting heat-tolerant accessions. 

Conclusively, the data generated here on a collection of SHWs represent a promising source for selecting heat-tolerant candidates for use in breeding, and the loci identified here can enhance our knowledge of the distribution of heat-tolerant alleles in wheat. 

## 4. Materials and Methods

### 4.1. Plant Material

A collection of 180 SHWs was used for this experiment (Table S1). These SHWs were derived from the combinations of 44 durum wheat cultivars and 100 *Ae. Tauschii* accessions as a subset of the panels previously characterized for grain morphology [44], biotic stress resistances [45,46], and grain quality [47].

### 4.2. Field Evaluation

To allow exposure of the genotypes to different heat treatments for the duration of flowering and ripening, six field tests were carried outat three different sites for 2 years (2014 and 2015), with two sowing time treatments (normal planting, termed as control; and late sowing, termed as heat stress (HS)). The three locations were the Regional Agricultural Research Institute at Bahawalpur in Punjab Province (BWP), the National Agricultural Research Centre (NARC) at Islamabad (ISB), and the Nuclear Institute of Agriculture at Tando Jam in Sindh (SND). The dates of normal sowing and late sowing were 15 November and 20 December, respectively. The sowing of genotypes was performed in two-row plots that were 2 m long and spaced 30 cm apart, with 15cm between rows, and in a randomized complete block design with three replications. The weight of seeds sown per plot was adjusted to obtain a constant seeding rate of 30 viable seeds per row by considering measurements of TKW and germination level. A small-plot grain drill was used for sowing purposes. Before plantation, triple superphosphate (4.3 g m^−2^ of P_2_O_5_) was applied by furrow placement, and urea was applied earlier than the second irrigation (8.6 g m^−2^ of N). During the crop season, irrigation was carried out four times at the following growth stages: (1) from germination to the appearance of seedlings, (2) from emergence to the double-ridge stage, (3) from double-ridge to anthesis, and (4) from anthesis to maturity [48]. The following traits were studied: plant height (PH, cm), measured as the distance from the soil level to the top of the spike, excluding awns; days to heading (DH); days to maturity (DM), considered as the number of days from sowinguntil the plant reached 50% maturity; grain number per square meter (GN), counted from 20 spikes randomly from every plot; tillers per plant (TpP), which were randomly selected from each genotype; spike length (SL) from the origin of the spike to the tip of the spike, without awns; spikelets per spike (SpSP), which were collected from each accession by picking three spikes and then counting the spikelets; weight of spikes per spike (SW), which was determined by weighing 3 spikes from each line; grain weight per spike (GWpS), which was calculated by measuring the weight of three randomly selected spikes from each row; thousand-kernel weight (TKW, g), which was measured after harvest by weighing double samples of 500 kernels from each plot; and grain yield (GY, kg m^−2^), which was calculated as the weight of grains produced per unit area. 

The heat susceptibility index (HSI) was calculated using the following formula: for example, for grain yield, HSIGY = (1 − Y/Yp)/D, where Y represents the yield of a genotype during the late planting, Yp shows the mean yield of genotypes at the time of normal planting, and stress intensity D = 1 − X/Xp, where X indicates the mean Y of all genotypes and Xp is the mean Yp of all genotypes.

### 4.3. Genotyping for the TaHST1Locus

The *TaHST1* locus was identified by using five primers: xhau1, xhau2, xhau3, xhau4, and xhau5. The primers’ details are listed in Table 6. The PCR reaction mixture (10 µL) contained PCR H_2_O (3 µL), master mix (5 µL), forward and reverse primers (0.5 µLeach), and DNA (1 µL). The conditions for PCR were as follows: initial denaturation at 95 °C for 5 min (1 cycle) or 94 °C for 1 min; annealing at 56 °C, 58 °C, 60 °C, and 65 °C for 1 min; and extension at 72 °C for 1 min (35 cycles); 2% agarose gel was used to check the PCR products.

### 4.4. DNA Extraction and Genotyping

Extraction of DNA was carried out using an amended CTAB method [49]. Synthetic wheat accessions were genotyped by using a 50 K SNP chip containing 66,798 SNPs. After filtering with a minor allele frequency of <5% and missing data of <20%, a total of 39,383 SNPs were then used for GWASs.

### 4.5. Statistical Analysis

#### 4.5.1. Analysis of Phenotypic Data

The phenotypic data were collected and arranged in Microsoft Excel. To test the statistical significance among different sources of variation, analysis of variance (ANOVA) was applied for all 11 traits. Phenotypic effects in the ANOVA model were divided into overall mean, genotypic effect, effect of random error, effect on replicate (i.e., block) in the environment (including both year and location), effect of environment, genotype with effect of treatment, genotype with environmental effect, and effect of treatment. Let *y*_lijk_ be the observed value of the trait of interest, where *i* is the accession and *k*is the replicate under the *j*th environmental condition along with the years and locations in this study, while *l* is the treatment. The linear model applied in ANOVA is as follows:

(1) $$y_{lijk}=\mu +D_{l}+R_{k/j}+G_{i}+E_{j}+GE_{ij}+GD_{il}+\varepsilon_{lijk}$$

where *l* = 1, 2, …, *L* (*L* = 2 for normal and heat stress treatments), *i* = 1, 2, …, *n* (*n* = 203), *j* = 1, 2, …, *e* (*e* = 6 with 3 locations and 2 years), *k* = 1, 2, …, *r* (*r* = 2) is the total mean value of the entire population, *R*_k/j_ is the *k*th effect on the replicate by the *j*th environment, *G*_i_ is the effect of the genotype on the *i*th accession, *E*_j_ is the effect of the environment on the *j*th environment, *GE*_ij_ is the effect of the interaction between the *i*th accession and the *j*th environment, *GD*_il_ is the effect of the interaction between the *i*th accession and the *l*th treatment, *ε*_lijk_ is the random error effect (which is supposed to be distributed normally, with a mean value of zero), and the variance is $\sigma_{\varepsilon }^{2}$. The ANOVA illustrated above was utilized in SAS software (SAS Institute, Cary, NC, USA, 2007) together with the GLM methods.

The genotypic values from the best linear unbiased prediction (BLUP) under normal and heat stress treatments for all of the accessions were considered as the subsequent phenotypes for comparison. The BLUP values were deliberated as follows: the trait experimental value was defined as *y*_ijk_, where *i* is the accession, *k* is the replicate, and *j* is the environment, with the locations and years used in this study. The BLUP values for the mixed model were determined as follows:

(2) $$y_{ijk}=\mu +R_{k/j}+G_{i}+E_{j}+GE_{ij}+\varepsilon_{ijk},\;\mathrm{and}\;\varepsilon_{ijk}\sim N(0,\sigma_{\varepsilon }^{2})$$

where *i* = 1, 2, …, *n* (*n* = 203), *j* = 1, 2, …, *e* (*e* = 6 by three locations and two years), *k* = 1, 2, …, and *r* (*r* = 2), *E_j_*,*GE_ij,_G_i_*, μ, and *R_k/j_*, are as described above. Otherwise, normal distribution was followed, and all of the effects were observed as random effects $R_{k/j}\sim N(0,\sigma_{R}^{2})$, $G_{i}\sim N(0,\sigma_{G}^{2})$, $E_{j}\sim N(0,\sigma_{E}^{2})$, and $GE_{ij}\sim N(0,\sigma_{GE}^{2})$, where $\sigma_{R}^{2}$, $\sigma_{G}^{2}$, $\sigma_{E}^{2}$, and $\sigma_{GE}^{2}$ are the variances representing the replicate, genotype, environment, and genotype–environment interaction, respectively. The values of BLUP were measured with the help of the MIXED procedure in the SAS software (SAS Institute, Cary, NC, USA, 2007). Pearson’s correlation was computed using the R program. 

#### 4.5.2. Genotypic Data Analysis and GWASs

GWASs for 11 phenotypic traits were conducted using a recently developed model selection algorithm—the Fixed and Random Model Circulating Probability Unification (FarmCPU; Liu et al., 2016) [50]. FarmCPU takes into account the confounding problem between covariates and test markers using a fixed-effects model (FEM) and a random-effects model (REM). The first five principal components calculated using TASSEL was used as covariates. The default *p*-value threshold that FarmCPU used was a Bonferroni-corrected threshold of 0.01. This Bonferroni-corrected threshold was overly restrictive when the LD among genotypic markers was large, so the threshold was calculated using the formula “*p* = 0.05/number of markers”, with 1000 permutations. In this function, the phenotypes were permuted to break the relationship with the genotypes. A vector of minimum *p*-values of each experiment was outputted, and the 95% quantile value of the vector was recommended for the *p* threshold in the FarmCPU model. The quantile–quantile (Q–Q) plot was used for assessing the fitness of the model to the population structure.

All of the SNPs associated with phenotypes under heat stress conditions were compared with the heat shock protein (HSP) framework of wheat [51], and those SNPs found within a ~5 Mb region of HSPs were reported to be candidate regions associated with *TaHSPs*.

Favorable and unfavorable alleles were identified for all marker–trait associations (MTAs). For each MTA, the allele with an increasing effect was deemed to be a favorable allele, while the alternate allele was deemed to be an unfavorable allele (i.e., allele with decreasing effect). For some traits, such as DH and PH, the allele with decreasing effect was deemed to be a favorable allele and the alternate allele was deemed to be an unfavorable allele (i.e., allele with increasing effect). In each accession, the frequency of favorable and unfavorable alleles was counted based on the number of trait-associated SNPs. The coefficient of determination (R^2^) was determined between the number of favorable/unfavorable alleles and the relevant phenotype as a measure of the combined allelic effects of all trait-associated SNPs on the phenotype. 

## Figures and Tables

**Table 1:** Descriptive statistics of synthetic hexaploid wheat under control and HS conditions.

Trait	Control			HS ^a^			HIS ^b^		
	Mean	Range	SD	Mean	Range	SD	Mean	Range	SD
DH	119.09	102–157	12.7	89.3	77–115	7.42	1	0.31–1.4	0.1
DM	156.5	139–186	7.7	115.5	75–148	9.11	1	0.55–1.97	0.1
TpP	13.67	7.3–20	2.4	7.28	4–11	1.4	0.97	−1.81	0.3
PH (cm)	111.8	19–140	16.9	79.9	56–103	8.9	0.89	−8–1.8	0.9
SL (cm)	15.8	3–25	2.8	9.6	6–14	1.2	0.93	−5.7–1.8	0.6
SpSP	13.7	4–23	3.1	19.7	11–28	3.6	1.15	−2.2–9.2	1.1
GpS	28.3	14–60	7.4	22.8	11–52	6.4	0.87	−8–5	1.3
SW (g)	2.1	0.7–3.9	0.96	1.8	0.9–3	0.3	1.69	−11–4.8	2.0
GWpS (g)	1.2	0.4–2.4	0.4	1.4	0.4–7	0.7	0.83	−2–10	2.2
GY (t/ha)	9.4	7–12	1.2	6.03	4–8	0.9	0.59	0.4–0.8	0.09
TKW (g)	46.9	19–68	8.2	36.3	18–49	4.7	0.94	−2.9–3.2	0.7

^a^ DH: days to heading; DM: days to maturity; TpP: tillers per plant; PH: plant height; SL: spike length; SpSP: spikelets per spike; GpS: grains per spike; SW: spike weight; GWpS: grain weight per spike; GY: grain yield; TKW: thousand-kernel weight. ^b^ HS: heat stress; HSI: heat susceptibility index.

**Table 2:** QTNs associated with phenotypic traits in control and heat stress (HS) conditions, with chromosome number, position, *p*-value, and minor allele frequency (MAF).

Traits	Condition	SNP	CHR	POS	Pos(Mb)	MAF	Effect	*p*-Value	Previous QTL/QTN
DH	HS	AX-109842766	1A	4.78 × 10^8^	477.74	0.20	3.16	7.23 × 10^−5^	
	HS	AX-111599726	3D	6.02 × 10^8^	602.27	0.19	3.67	2.36 × 10^−5^	
	HSI	AX-179559172	4B	6.50 × 10^8^	649.6	0.17	0.09	8.18 × 10^−5^	
	HS	AX-94503623	5B	6.19 × 10^8^	619.14	0.24	3.46	5.61 × 10^−5^	
	HS	AX-86180088	6A	2.71 × 10^8^	270.61	0.16	3.50	7.96 × 10^−5^	
	HS	AX-95660535	6A	3.15 × 10^8^	315.41	0.17	3.60	4.87 × 10^−5^	
	HS	AX-94744160	6A	3.81 × 10^8^	381.03	0.16	3.50	7.96 × 10^−5^	
	HS	AX-109950287	6A	3.90 × 10^8^	390.08	0.16	3.70	3.85 × 10^−5^	
	Control	AX-94444292	6B	9.78 × 10^7^	97.82	0.05	−11.18	6.24 × 10^−5^	
	HSI	AX-89339930	6B	4.62 × 10^8^	462.15	0.20	0.08	1.50 × 10^−5^	
	HSI	AX-95169568	6B	7.04 × 10^8^	703.94	0.24	0.08	2.91 × 10^−5^	
	HSI	AX-86173990	6D	4.58 × 10^8^	458.36	0.19	0.08	5.48 × 10^−5^	
	HSI	AX-110389623	6D	4.63 × 10^8^	462.7	0.25	0.08	9.35 × 10^−5^	
	Control	AX-95659681	7A	7.21 × 10^8^	721.22	0.39	7.79	8.04 × 10^−5^	
	HS	AX-94980563	7B	4.38 × 10^8^	437.93	0.13	4.08	1.97 × 10^−5^	
	HSI	AX-94750097	7D	6.24 × 10^8^	623.54	0.23	0.10	7.49 × 10^−5^	
DM	HSI	AX-94754683	2B	6.71 × 10^6^	6.71	0.05	0.13	9.39 × 10^−5^	
	Control	AX-94584932	2B	3.22 × 10^8^	321.96	0.31	2.55	4.77 × 10^−4^	
	Control	AX-95660688	3B	1.59 × 10^6^	1.59	0.37	2.37	8.98 × 10^−4^	
	Control	AX-109430132	3B	6.29 × 10^8^	628.8	0.14	3.16	8.93 × 10^−4^	
	HS	AX-94786898	3D	5.88 × 10^8^	588.33	0.07	−6.77	5.83 × 10^−5^	
	Control	AX-94457757	4B	6.53 × 10^8^	652.98	0.10	5.59	5.61 × 10^−4^	
	Control	AX-110458863	4D	4.28 × 10^7^	42.81	0.11	3.63	6.95 × 10^−4^	
	Control	AX-94605765	6A	8.50 × 10^7^	84.98	0.31	2.67	2.94 × 10^−4^	
	Control	AX-86180088	6A	2.71 × 10^8^	270.61	0.16	2.96	6.43 × 10^−4^	
	HS	AX-110984345	6A	3.85 × 10^8^	384.73	0.15	4.75	6.89 × 10^−5^	
	Control	AX-109950287	6A	3.90 × 10^8^	390.08	0.16	3.11	4.03 × 10^−4^	
	HS	AX-109950287	6A	3.90 × 10^8^	390.08	0.16	4.59	8.56 × 10^−5^	
	Control	AX-109886100	6B	1.41 × 10^8^	140.62	0.32	2.48	5.44 × 10^−4^	
	Control	AX-109397401	6D	8.03 × 10^5^	0.8	0.05	5.04	6.60 × 10^−4^	
	Control	AX-94980563	7B	4.38 × 10^8^	437.93	0.13	3.27	4.85 × 10^−4^	
	HS	AX-94980563	7B	4.38 × 10^8^	437.93	0.13	5.25	2.36 × 10^−5^	
	Control	AX-95658771	7B	6.09 × 10^8^	608.72	0.27	3.48	4.71 × 10^−4^	
	Control	AX-94479139	7D	5.07 × 10^8^	507.25	0.15	5.26	3.18 × 10^−4^	
GpS	HS	AX-95150128	1D	7.91 × 10^7^	79.14	0.11	−3.19	7.32 × 10^−4^	
	HS	AX-110335177	1D	3.95 × 10^8^	394.73	0.09	3.49	6.74 × 10^−4^	
	HS	AX-110911350	2A	4.42 × 10^7^	44.18	0.12	2.85	7.46 × 10^−4^	
	HS	AX-110046675	2A	4.47 × 10^7^	44.75	0.13	2.85	7.46 × 10^−4^	
	HS	AX-110533769	2B	7.17 × 10^8^	717.22	0.21	2.50	6.62 × 10^−4^	
	HS	AX-179562568	2D	5.99 × 10^8^	598.71	0.10	2.97	9.43 × 10^−4^	
	HS	AX-94942177	3A	4.75 × 10^7^	47.55	0.43	2.77	2.66 × 10^−4^	
	Control	AX-95244621	3A	7.32 × 10^8^	732.24	0.41	2.45	6.90 × 10^−4^	
	HS	AX-109270732	3B	6.37 × 10^8^	637.08	0.09	4.10	1.04 × 10^−4^	
	Control	AX-89477157	3B	8.23 × 10^8^	822.97	0.33	2.83	5.42 × 10^−4^	
	Control	AX-94710887	3D	6.14 × 10^8^	613.74	0.34	2.83	5.42 × 10^−4^	
	Control	AX-109290547	4A	7.13 × 10^8^	712.92	0.13	3.23	7.51 × 10^−4^	
	Control	AX-110396807	4B	4.49 × 10^7^	44.94	0.47	−2.40	4.71 × 10^−4^	
	Control	AX-95094806	4D	3.12 × 10^8^	311.96	0.30	2.88	6.76 × 10^−4^	
	Control	AX-95253860	4D	5.00 × 10^8^	499.62	0.38	2.25	9.17 × 10^−4^	
	HSI	AX-94948850	5B	4.26 × 10^8^	426.09	0.05	−1.44	7.86 × 10^−5^	
	HS	AX-110559492	5B	5.91 × 10^8^	591.14	0.46	2.07	6.79 × 10^−4^	MQTL5B.2
	HSI	AX-110136588	5B	6.50 × 10^8^	650.08	0.11	0.65	9.19 × 10^−5^	
	Control	AX-111160156	6A	1.66 × 10^7^	16.57	0.22	2.72	5.36 × 10^−4^	
	Control	AX-179558749	6A	1.01 × 10^8^	101.3	0.10	−3.69	6.05 × 10^−4^	
	Control	AX-94729591	6B	2.82 × 10^7^	28.16	0.24	2.61	9.31 × 10^−4^	
	Control	AX-112289907	6B	4.11 × 10^8^	411.1	0.38	2.72	4.17 × 10^−4^	
	HS	AX-95653657	7B	5.96 × 10^7^	59.61	0.18	−3.00	7.08 × 10^−4^	
	Control	AX-94624899	7B	7.09 × 10^8^	709.38	0.37	−2.53	4.15 × 10^−4^	
GWpS	HSI	AX-110932792	1A	5.66 × 10^8^	566.01	0.12	1.40	1.81 × 10^−5^	
	HSI	AX-95654468	1B	1.43 × 10^8^	142.52	0.06	2.70	1.43 × 10^−5^	
	HS	AX-95633859	1D	3.57 × 10^8^	357.46	0.07	0.51	8.39 × 10^−5^	
	Control	AX-94725049	2A	3.45 × 10^6^	3.45	0.08	−0.27	3.26 × 10^−5^	
	Control	AX-109388159	2B	6.18 × 10^6^	6.18	0.16	−0.20	8.21 × 10^−5^	
	HS	AX-94648480	2B	1.88 × 10^8^	188.02	0.10	0.48	7.50 × 10^−5^	
	HS	AX-94997695	2B	1.98 × 10^8^	198.07	0.10	0.48	7.50 × 10^−5^	
	HS	AX-95202875	3A	5.72 × 10^8^	571.79	0.06	0.66	3.93 × 10^−5^	
	HSI	AX-108804725	3A	7.13 × 10^8^	713.21	0.09	1.49	9.30 × 10^−5^	
	HS	AX-109911103	5A	6.12 × 10^8^	611.65	0.08	0.51	6.49 × 10^−5^	
	HS	AX-94886530	5B	1.87 × 10^8^	186.84	0.05	0.61	6.92 × 10^−5^	
	HS	AX-111441228	5D	5.49 × 10^8^	548.64	0.08	0.48	8.07 × 10^−5^	
	HS	AX-94846296	6A	7.40 × 10^7^	74.03	0.05	0.58	8.93 × 10^−5^	
	HS	AX-94746774	6A	9.64 × 10^7^	96.44	0.05	0.58	8.93 × 10^−5^	
	HS	AX-110087041	6B	1.32 × 10^8^	132.12	0.05	0.58	8.93 × 10^−5^	
	HS	AX-95685124	6B	1.52 × 10^8^	152.09	0.05	0.58	8.93 × 10^−5^	
	HS	AX-179476704	6D	6.21 × 10^7^	62.11	0.08	0.51	6.93 × 10^−5^	
GY	HS	AX-94580758	1B	6.27 × 10^8^	626.6	0.50	0.34	3.32 × 10^−4^	MQTL1B.4
	HS	AX-111214234	1D	4.56 × 10^8^	455.8	0.29	−0.39	2.25 × 10^−4^	MQTL1D.5
	HSI	AX-94869969	2B	3.87 × 10^8^	387.13	0.21	0.06	6.36 × 10^−5^	
	HSI	AX-95230131	2B	6.38 × 10^8^	638.26	0.19	0.05	8.41 × 10^−5^	MQTL2B.3, MQTL2B.6, MQTL2B.7
	HSI	AX-94411868	2D	5.38 × 10^8^	537.51	0.23	0.05	4.16 × 10^−5^	
	HS	AX-111599726	3D	6.02 × 10^8^	602.27	0.19	−0.45	3.26 × 10^−4^	
	Control	AX-94752113	4A	1.38 × 10^7^	13.76	0.38	0.60	9.72 × 10^−5^	
	Control	AX-94525682	4B	5.64 × 10^8^	564.22	0.39	0.59	8.24 × 10^−5^	
	HSI	AX-108840130	4B	6.22 × 10^8^	621.71	0.15	0.05	3.17 × 10^−5^	
	Control	AX-94988232	4D	4.56 × 10^8^	455.75	0.40	0.59	8.24 × 10^−5^	
	HSI	AX-94882874	4D	4.87 × 10^8^	487.05	0.17	0.05	4.94 × 10^−5^	
	HSI	AX-94423826	5B	5.89 × 10^8^	588.83	0.10	−0.06	2.89 × 10^−5^	
	HSI	AX-111052660	5B	6.11 × 10^8^	610.88	0.30	0.04	6.44 × 10^−5^	
	HSI	AX-95651957	6D	2.41 × 10^6^	2.41	0.50	0.04	5.17 × 10^−5^	MQTL6D.1
	HS	AX-111558280	6D	4.64 × 10^8^	463.79	0.45	0.35	4.26 × 10^−4^	
	HS	AX-111603870	7D	4.64 × 10^6^	4.64	0.39	−0.33	6.79 × 10^−4^	
PH	Control	AX-111780688	1B	5.30 × 10^8^	529.99	0.17	−8.14	7.10 × 10^−5^	MQTL1B.5
	Control	AX-95235626	2A	6.28 × 10^8^	627.51	0.11	−10.21	3.84 × 10^−5^	
	HS	AX-89556873	2B	7.32 × 10^8^	732.05	0.11	−5.49	7.58 × 10^−5^	MQTL2B.2
	HS	AX-94512274	3A	5.61 × 10^7^	56.09	0.20	4.36	3.45 × 10^−5^	
	HS	AX-95237322	3D	4.50 × 10^7^	45.02	0.17	4.67	1.88 × 10^−5^	
	Control	AX-95094658	5B	3.98 × 10^8^	398.08	0.08	−11.72	3.16 × 10^−5^	
	Control	AX-89381684	5B	4.79 × 10^8^	478.76	0.09	−11.58	1.35 × 10^−5^	MQTL5B.2
	HSI	AX-86179657	5B	6.70 × 10^8^	670.08	0.13	−0.61	4.43 × 10^−5^	
	HSI	AX-108844431	5D	3.24 × 10^7^	32.44	0.10	−0.59	5.70 × 10^−5^	
	HS	AX-110726038	6D	5.73 × 10^5^	0.57	0.08	6.11	8.46 × 10^−5^	
	HSI	AX-109431581	6D	2.34 × 10^8^	23.44	0.13	−0.55	6.46 × 10^−5^	
	HSI	AX-86177729	6D	4.62 × 10^8^	461.71	0.13	−0.57	3.57 × 10^−5^	
	HSI	AX-110911240	7D	5.50 × 10^8^	549.75	0.12	−0.54	4.38 × 10^−5^	
SL	Control	AX-179559739	2D	6.36 × 10^7^	63.57	0.50	1.12	7.83 × 10^−5^	MQTL2D.1
	Control	AX-109809781	2D	6.06 × 10^8^	606.12	0.10	1.79	4.89 × 10^−5^	
	Control	AX-94522134	2D	6.29 × 10^8^	629	0.26	1.39	1.34 × 10^−5^	
	HSI	AX-94959965	3A	7.28 × 10^8^	727.99	0.19	−0.47	1.72 × 10^−5^	
	HSI	AX-111016736	3B	7.74 × 10^8^	773.76	0.08	−0.51	8.92 × 10^−5^	
	HS	AX-179557838	3B	8.22 × 10^8^	822.19	0.44	0.47	6.78 × 10^−5^	
	HSI	AX-94752943	4A	6.04 × 10^8^	603.68	0.06	−0.49	7.20 × 10^−5^	
SpPS	HS	AX-179388661	1A	5.94 × 10^8^	593.55	0.33	−1.39	7.02 × 10^−4^	
	Control	AX-86174804	1D	7.87 × 10^6^	7.87	0.11	1.35	9.64 × 10^−4^	
	HS	AX-109545057	2B	5.39 × 10^8^	539.33	0.19	−2.19	8.81 × 10^−4^	
	HS	AX-94415873	2D	5.51 × 10^8^	550.65	0.24	−1.30	5.48 × 10^−4^	
	HSI	AX-94669552	5B	4.42 × 10^7^	44.23	0.12	0.71	6.21 × 10^−5^	
	HSI	AX-95629141	5B	5.66 × 10^7^	56.56	0.09	0.79	6.39 × 10^−5^	
SW	Control	AX-179388053	2B	1.76 × 10^8^	176.29	0.45	−0.42	6.52 × 10^−5^	
	HSI	AX-109809781	2D	6.06 × 10^8^	606.12	0.10	−1.48	2.30 × 10^−5^	
	HSI	AX-109312408	3D	6.08 × 10^8^	608.25	0.09	−1.41	7.84 × 10^−5^	
	HSI	AX-94700952	4B	6.12 × 10^8^	611.51	0.14	−1.23	4.88 × 10^−5^	
	HS	AX-110516900	4B	6.70 × 10^8^	669.71	0.07	−0.24	1.32 × 10^−5^	
	HS	AX-109482775	4B	6.70 × 10^8^	670.43	0.07	−0.24	1.32 × 10^−5^	
	HS	AX-108778179	6B	5.91 × 10^8^	591.21	0.14	−0.17	4.79 × 10^−5^	
	HSI	AX-111907482	6D	4.69 × 10^8^	469.1	0.05	−2.12	2.75 × 10^−5^	
TKW	Control	AX-110402508	2A	4.47 × 10^7^	44.73	0.11	−4.28	9.55 × 10^−4^	
	Control	AX-108895787	2A	4.51 × 10^7^	45.07	0.12	−4.37	5.74 × 10^−4^	
	Control	AX-108944653	2D	6.00 × 10^8^	599.89	0.21	−3.36	6.55 × 10^−4^	
	Control	AX-109948996	2D	6.10 × 10^8^	609.64	0.18	−4.42	3.01 × 10^−4^	
	Control	AX-109551920	3A	7.28 × 10^8^	727.99	0.08	−9.31	1.60 × 10^−4^	
	Control	AX-110589570	3B	6.25 × 10^8^	625.42	0.15	−4.74	2.41 × 10^−4^	
	HSI	AX-109270732	3B	6.37 × 10^8^	637.08	0.09	−0.47	8.26 × 10^−5^	
	HS	AX-94460648	6D	4.62 × 10^8^	462.36	0.49	−1.84	6.99 × 10^−5^	
TpP	Control	AX-95654322	1A	5.80 × 10^8^	580.5	0.10	2.24	8.94 × 10^−5^	
	HSI	AX-109016685	2A	1.91 × 10^7^	19.11	0.20	0.13	4.13 × 10^−4^	
	HSI	AX-95093243	2A	7.72 × 10^8^	771.51	0.08	0.14	9.39 × 10^−4^	
	HS	AX-94760904	2B	7.31 × 10^8^	731.35	0.33	−0.60	3.21 × 10^−5^	
	HSI	AX-179477221	6A	2.31 × 10^8^	230.7	0.32	0.11	3.02 × 10^−4^	
	HSI	AX-179475799	6A	3.59 × 10^8^	359.08	0.08	0.23	6.18 × 10^−4^	
	HSI	AX-95016643	6A	3.85 × 10^8^	384.68	0.06	0.24	8.94 × 10^−4^	
	HSI	AX-95119810	6A	3.87 × 10^8^	387.07	0.16	0.11	6.18 × 10^−4^	
	HSI	AX-94949800	7B	4.72 × 10^8^	472.33	0.08	0.17	5.75 × 10^−4^	
	HSI	AX-94665491	7D	1.51 × 10^8^	151.32	0.33	0.12	2.70 × 10^−4^	

Abbreviations: DH: days to heading; DM: days to maturity; TpP: tillers per plant; PH: plant height; SL: spike length; SpSP: spikelets per spike; GpS: grains per spike; SW: spike weight; GWpS: grain weight per spike; GY: grain yield; TKW: thousand-kernel weight. All previous QTLs are based on the work of Tanin et al. [21].

**Table 3:** Pleiotropic QTNs associated with various yield traits under control, heat susceptibility index (HSI) and heat stress (HS) conditions, with chromosome number, position (Mb), effect, and *p*-value.

Condition	Traits	SNP	CHR	Pos (Mb)	Effect	*p*-Value
HS	GpS	AX-110911350	2A	44.18	2.85	7.46 × 10^−4^
Control	TKW	AX-110402508	2A	44.73	−4.28	9.55 × 10^−4^
HS	GpS	AX-110046675	2A	44.75	2.85	7.46 × 10^−4^
Control	TKW	AX-108895787	2A	45.07	−4.37	5.74 × 10^−4^
Control	GWpS	AX-109388159	2B	6.18	−0.20	8.21 × 10^−5^
HSI	DM	AX-94754683	2B	6.71	0.13	9.39 × 10^−5^
HS	TpP	AX-94760904	2B	731.35	−0.60	3.21 × 10^−5^
HS	PH	AX-89556873	2B	732.05	−5.49	7.58 × 10^−5^
HS	GpS	AX-179562568	2D	598.71	2.97	9.43 × 10^−4^
Control	TKW	AX-108944653	2D	599.89	−3.36	6.55 × 10^−4^
HSI	SL	AX-94959965	3A	727.99	−0.47	1.72 × 10^−5^
Control	TKW	AX-109551920	3A	727.99	−9.31	1.60 × 10^−4^
Control	TKW	AX-110589570	3B	625.42	−4.74	2.41 × 10^−4^
Control	DM	AX-109430132	3B	628.80	3.16	8.93 × 10^−4^
HS	GpS	AX-109270732	3B	637.08	4.10	1.04 × 10^−4^
HSI	TKW	AX-109270732	3B	637.08	−0.47	8.26 × 10^−5^
HS	SL	AX-179557838	3B	822.19	0.47	6.78 × 10^−5^
Control	GpS	AX-89477157	3B	822.97	2.83	5.42 × 10^−4^
HS	DH	AX-111599726	3D	602.27	3.67	2.36 × 10^−5^
HS	GY	AX-111599726	3D	602.27	−0.45	3.26 × 10^−4^
HIS	GY	AX-94423826	5B	588.83	−0.06	2.89 × 10^−5^
HS	GpS	AX-110559492	5B	591.14	2.07	6.79 × 10^−4^
HS	DH	AX-86180088	6A	270.61	3.50	7.96 × 10^−5^
Control	DM	AX-86180088	6A	270.61	2.96	6.43 × 10^−4^
HS	DH	AX-94744160	6A	381.03	3.50	7.96 × 10^−5^
HSI	TpP	AX-95016643	6A	384.68	0.24	8.94 × 10^−4^
HS	DM	AX-110984345	6A	384.73	4.75	6.89 × 10^−5^
HS	DH	AX-109950287	6A	390.08	3.70	3.85 × 10^−5^
Control	DM	AX-109950287	6A	390.08	3.11	4.03 × 10^−4^
HSI	DH	AX-86173990	6D	458.36	0.08	5.48137 × 10^−5^
HSI	PH	AX-86177729	6D	461.71	−0.57	3.57 × 10^−5^
HS	TKW	AX-94460648	6D	462.36	−1.84	6.99 × 10^−5^
HSI	DH	AX-110389623	6D	462.70	0.08	9.35026 × 10^−5^
HS	GY	AX-111558280	6D	463.79	0.35	4.26 × 10^−4^
HS	DH	AX-94980563	7B	437.93	4.08	1.97 × 10^−5^
Control	DM	AX-94980563	7B	437.93	3.27	4.85 × 10^−4^

Abbreviations: DH: days to heading; DM: days to maturity; TpP: tillers per plant; PH: plant height; SL: spike length; SpSP: spikelets per spike; GpS: grains per spike; SW: spike weight; GWpS: grain weight per spike; GY: grain yield; TKW: thousand-kernel weight.

**Table 4:** QTNs associated with heat stress conditions and in proximity to heat shock proteins, with chromosome number, position (Mb), and position of closest HSP.

Traits	SNP	CHR	Pos(Mb)	Hsp	Gene ID	POS(Mb)2
GpS	AX-110533769	2B	717.22	TaHSP40.54	TraesCS2B01G526300	720.58
TpP	AX-94760904	2B	731.35	TaHSP70.24	TraesCS2B01G535000	731.25
PH	AX-94512274	3A	56.09	TaHSP40.63	TraesCS3A01G083700	53.83
PH	AX-95237322	3D	45.02	TaHSP40.95	TraesCS3D01G083700	42.49
SW	AX-109482775	4B	670.43	TaHSP70.52	TraesCS4B01G397600	671.74
GWpS	AX-109911103	5A	611.65	TaHSP40.150	TraesCS5A01G426100	611.34
GWpS	AX-94886530	5B	186.84	TaHSP40.155	TraesCS5B01G114500	185.72
TKW	AX-94460648	6D	462.36	TaHSP60.69	TraesCS6D01G383500	462.46

CHR: chromosome; Pos (Mb): position of SNP; Hsp: heat shock protein ID; GeneID: Gene ID of heat shock protein according to IWGSC; POS(MB)2: position of heat shock protein encoding the gene.

**Table 5:** Description of haplotypes for the *TaHST1* locus, including frequency (%) in the diversity panel of synthetic hexaploid wheats.

Haplotype	Xhau1	Xhau2	Xhau3	Xhau4	Xhau5	Deleted Sites	No. of Lines	Frequency %
Hap1	+	+	+	127	+	0	33	25
Hap2		-	-	127	+	3	21	16
Hap3	-	+	+	127	+	1	20	15
Hap4	-	+	+	127	-	2	13	10
Hap5	-	-	-	127	-	4	13	10
Hap6	+	+	+	127	-	1	10	8
Hap7	-	-	+	127	+	2	6	5
Hap8	-	-	-	195	-	4	5	4
Hap9	-	+	-	127	+	2	3	2
Hap10	+	+	-	127	+	1	2	2
Hap11	-	-	-	195	+	3	2	2
Hap12	-	-	-	-	-	5	1	1
Hap13	+	+	-	127	-	2	1	1
Hap14	+	-	+	127	+	1	1	1
Hap15	-	-	+	195	+	2	1	1
Total							132	100

Note: the + and - signs show positive and negative amplification. Amplification of 127 bp and 195 bp was performed using Xhau-4, due to its co-dominant nature.

**Table 6:** Primers used in the study for the identification of allelic variations at *TaHST1* associated with HST in synthetic wheat.

Primer	Sequence	T_m_ °C	Product Size (bp)
Xhau1-F	GGGAGTGTTTGTGTGAGGATTTG	65	825
Xhau1-R	GCACTACTACCAAACCACGTGTA		
Xhau2-F	GGGAGCCAATTCGTGTGACT	56	426
Xhau2-R	CAAGCGCTATACAACTGTGCT		
Xhau3-F	GCCCGTGAATCATACTTGAGCG	58	788
Xhau3-R	TGAGGAGATAATTGTACGCCGA		
Xhau4-F	TCGGTTGGTTTGTTTATACTTGC	65	127/195
Xhau4-R	CCACGCTTGCACAATCTATTCT		
Xhau5-F	TGCCTACCAAAGTGAGACCTG	60	259
Xhau5-R	ACCTACCTCTACCTCAACCCA		

## Data Availability

All of the phenotypic and genotypic data used in this study are shared in the Supplementary Materials.

## Supplementary Materials

The following supporting information can be downloaded at https://www.mdpi.com/article/10.3390/plants12081610/s1: Supplementary Table S1: Names and pedigree of the 180 genotypes used in this study; Supplementary Table S2: Phenotypic data on synthetic hexaploid wheats in control and heat stress treatments; Table S3a: Meteorological information of Bahawalpur (BWP) for normal planting and late planting during the two cropping seasons (2013/14 and 2014/15); Table S3b: Meteorological information of Sindh (SND) for normal planting and late planting during the two cropping seasons (2013/14 and 2014/15); Table S3c: Meteorological information of Islamabad (ISD) for normal planting and late planting during the two cropping seasons (2013/14 and 2014/15); Supplementary Table S4: Phenotypic data on synthetic hexaploid wheats in control and heat stress treatments; Supplementary Table S5: Genotypic data as a HapMap file of all synthetic hexaploid wheats used in this study; Figure S1: Manhattan and quantile–quantile (Q-Q) plots of various phenotypic traits, including DH (A), TKW (B), TpP (C), PH (D), SL (E), SpSP (F), GpS (G), SW (H), GWpS (I), and GY (H); Manhattan plots using BLUP values.
