# Supplementary material for: Identification of Single-Nucleotide Polymorphisms (SNPs) Associated with Heat Tolerance at the Reproductive Stage in Synthetic Hexaploid Wheats Using GWAS
Source: Plants (Basel). 2023 Apr 10;12(8):1610. doi: 10.3390/plants12081610 (PMC10142051; doi:10.3390/plants12081610)
Supplement: Supplementary file 1 [file plants-12-01610-s001.zip › FIGURE S1.pdf]

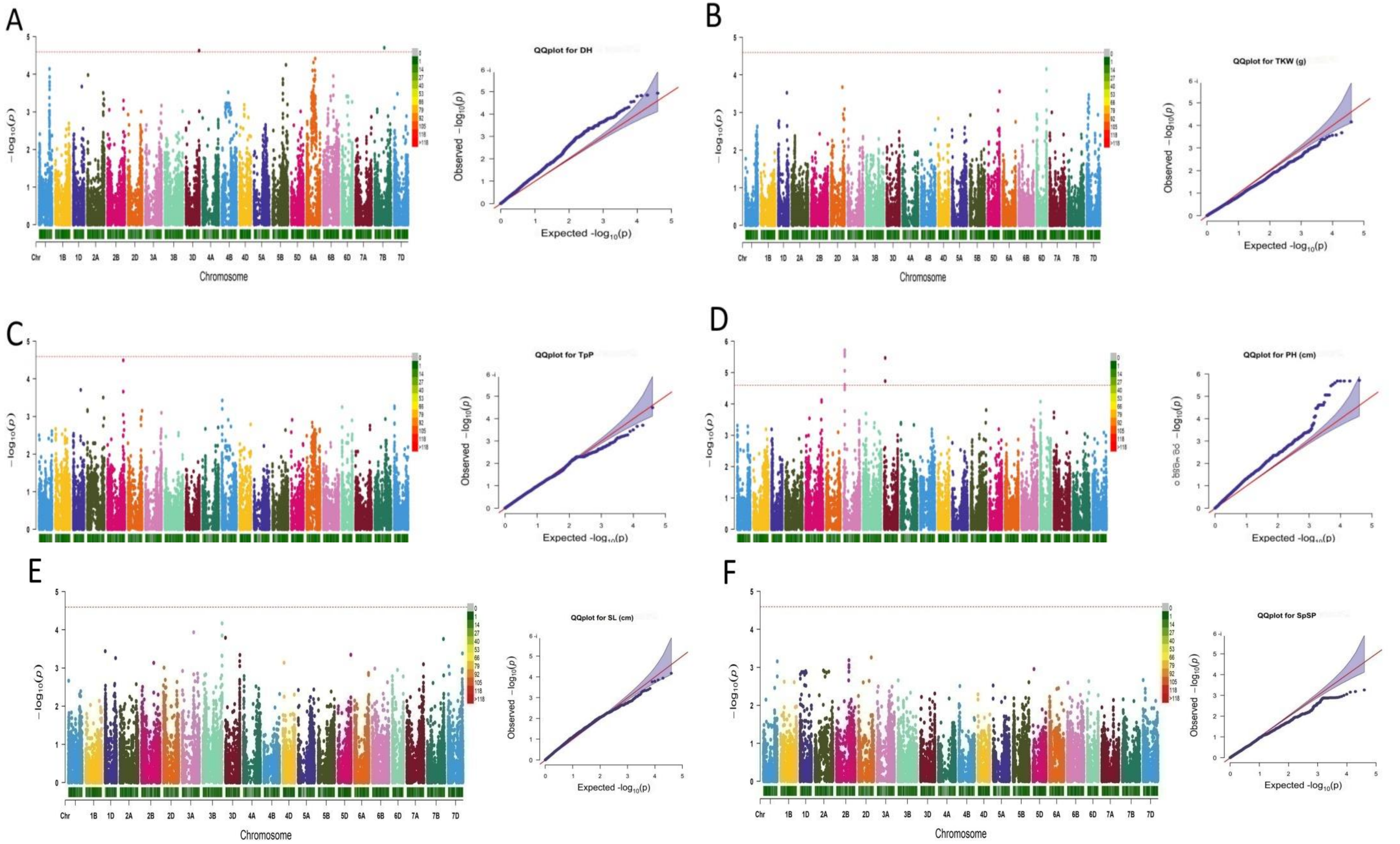
A
QQplot for DH
B
QQplot for TKW (g)
C
QQplot for TpP
D
QQplot for PH (cm)
E
QQplot for SL (cm)
F
QQplot for SpSP
$-\log_{10}(p)$
Observed $-\log_{10}(p)$
Expected $-\log_{10}(p)$
Chromosome
Chr 1B 1D 2A 2B 2D 3A 3B 3D 4A 4B 4D 5A 5B 5D 6A 6B 6D 7A 7B 7D

Continue…

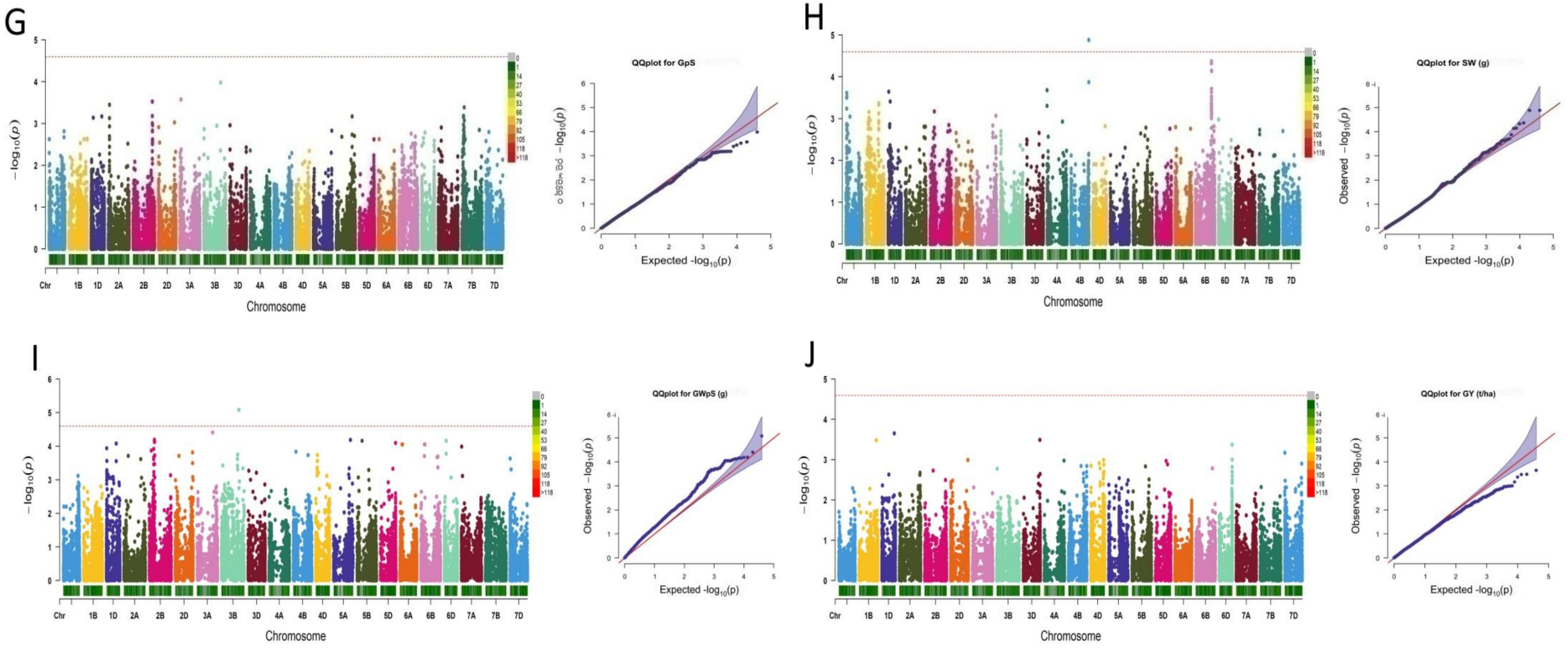

Figure S1. Manhattan and quantile-quantile (QQ) plots of various phenotypic traits including DH (A), TKW (B), TpP (C), PH (D), SL (E), SpSP (F), GpS (G), SW (H), GWpS (I) and GY (H) using BLUP values.

Abbreviations: DH: days to heading; DM: days to maturity; TpP: Tillers per plant; PH: Plant height; SL: Spike length; SpSP: Spikelets per spike; GpS: Grains per spike; SW: Spike weight; GWpS: Grain weight per spike; GY: Grain yield; TKW: Thousand kernel weight
